# Malaria and Typhoid Fever Coinfection among Febrile Patients in Ngaoundéré (Adamawa, Cameroon): A Cross-Sectional Study

**DOI:** 10.1155/2023/5334813

**Published:** 2023-09-25

**Authors:** Francky Steve Sohanang Nodem, Didiane Ymele, Mouni Fadimatou, Simeon-Pierre Chegaing Fodouop

**Affiliations:** ^1^Department of Food Engineering and Quality Control, University Institute of Technology, University of Ngaoudéré, P.O. Box 455, Ngaoundéré, Cameroon; ^2^Department of Biomedical Sciences, Faculty of Sciences, University of Ngaoundéré, P.O. Box 455, Ngaoundéré, Cameroon

## Abstract

**Background:**

Malaria and typhoid fever remain the major cause of morbidity and mortality in tropical and subtropical countries. It is common today to see patients being concurrently infected or treated for the two diseases.

**Objective:**

The aim of the present study was to determine the prevalence of malaria, typhoid fever, and their coinfection among febrile patients at Ngaoundéré Regional Hospital, Adamawa, Cameroon. *Methodology*. A cross-sectional and descriptive study was conducted on 208 febrile patients coming for medical consultation at Ngaoundéré Regional Hospital and suspected for malaria and/or typhoid fever from September to November 2019. After receiving the consent of each patient, clinical and sociodemographic information were collected using a pretested semistructured questionnaire. Thereafter, blood samples were collected and subjected to (i) blood film examination for malaria detection and (ii) qualitative and semiquantitative Widal assay for typhoid fever detection.

**Results:**

*Plasmodium falciparum* was the only species identified, and the prevalence of malaria was 50.0% (*n* = 104). The seroprevalence of typhoid fever was 64.3% (*n* = 133). The malaria and typhoid fever coinfection was 30.3% (*n* = 63). The coinfection between *S. paratyphi* (A, B, and C) and malaria was also observed with the prevalence ranging from 32.2 to 63.9%. Female group and children from 2 to 10 years old were the most affected groups by the two infectious agents.

**Conclusion:**

Typhoid fever and malaria were more prevalent in Ngaoundéré City, particularly in children and women. Further studies should be done on the risk factors of malaria and typhoid fever coinfection in different study areas.

## 1. Introduction

Malaria is a life-threatening disease caused by protozoan parasites belonging to genus *Plasmodium* that are transmitted through the bites of infected female *Anopheles* mosquitoes [1–4]. This disease causes widespread deaths to children and economic hardships on poor household families [2, 3, 5]. Malaria causes symptoms that typically include fever, fatigue, vomiting, and headaches. In severe cases, it can cause yellow skin, seizures, coma, or death [1]. In the year 2018, WHO estimates that there were about 228 million cases of malaria and 405,000 malaria-related deaths with 93.8% of deaths occurring in sub-Saharan Africa [6, 7]. The majority of infections are caused by *Plasmodium falciparum* species, the most dangerous of the four human malaria parasites [7, 8].

Typhoid fever is an acute systemic infection caused by *Salmonella typhi*, a Gram and oxidase-negative bacterium [1]. It is mainly transmitted faeco-orally through contaminated food and water. There are about 33 million cases of typhoid annually in the world resulting in 216,000 deaths in endemic areas [1]. Outbreaks of typhoid fever are frequently reported from sub-Saharan Africa and countries in Southeast Asia [1]. WHO identifies typhoid as a serious public health problem with high incidence on children, young adults, and pregnant women [1, 3]. The most prominent feature of the infection is fever, which gradually rises to a high value. High fever, lethargy, skin rash, loss of appetite, constipation more than often, abdominal pain, diarrhea, hepatosplenomegaly, bradycardia, and headaches are the commonest symptoms, and without treatment, symptoms may last for weeks or months [8, 9].

Both diseases, mainly associated with poverty and underdevelopment with significant morbidity and mortality, are common in many countries where the prevailing environmental conditions of warm humid climate, poor sanitary habits, poverty, and ignorance exist [6]. In addition, malaria and typhoid fever coinfection studies have already been carried out in Africa and specifically in Nigeria [6–8], Zambia [10], Burkina Faso [11], and Cameroon [12–14]. Despite the already existing insufficient information on concurrent malaria and typhoid coinfection in Cameroon, information on this association is still lacking in rural communities of Cameroon. The aim of this study was to update the basic descriptive epidemiological data on the malaria and typhoid fever coinfection among the population of Ngaoundéré, Cameroon.

## 2. Materials and Methods

### 2.1. Study Site

The study was conducted from September to November 2019 at the Ngaoundéré Regional Hospital, a reference hospital and the most frequented in the Adamawa Region of Cameroon. The region has a population of about 1.18 million, an area of 63,701 km^2^ that borders on the west with Nigeria and on the east with the Central African Republic [4]. The climate is temperate, and its savannah vegetation is situated in a hilly area. The region is under the influence of both a dry season (November-April) and a rainy season (May-October). Its mean annual temperature lies between 20 and 25°C, while mean annual rainfall ranges from 900 mm to 1500 mm [15]. The region has several displaced camps for refugees or displaced people coming from neighbouring countries, such as Nigeria, Chad, or the Central African Republic [4]. All subjects coming to the Ngaoundéré Regional Hospital and suspected for malaria and/or typhoid fever were included in the study (*N* = 208).

### 2.2. Biodata of the Participants and Blood Collection

The study population comprises febrile patients coming for medical consultation at Ngaoundéré Regional Hospital and suspected for malaria and/or typhoid fever from September to November 2019. They were recruited by voluntary participation, and information were collected using a pretested structured questionnaire. Informed consents were prior obtained from all participants before selection. Patients were free to refuse to answer any question if they choose to do so. Data on the sociodemographic (i.e., age, sex, residence, educational level, occupation, and marital status), clinical characteristics (i.e., fever, headache, joint pain, fatigue, chill, vomiting, and diarrhea), and treatment approach or medical history (antimalarial or antibiotics used, traditional medicine application) of the participants were collected using a pretested structured questionnaire by interview. After the interview, 4–5 mL blood sample was collected from different patients into sterilized EDTA® falcon tubes by trained phlebotomist.

### 2.3. Sample Analysis

#### 2.3.1. Thick Blood Film for Malaria Parasite Detection

Diagnosis of malaria was done by the microscopic examination (×100 objective) of stained thick and thin blood films. The blood films (thick and thin) were stained with 10% rapid Giemsa (SD Bioline, Inc., USA) for 15 minutes for the detection of *Plasmodium* parasites and speciation, respectively. The parasitemia was evaluated by counting the number of parasites against 200 white blood cells on thick films assuming a total leucocyte count of 8000/*μ*L of blood. At least 100 high-power microscopic fields were examined before declaring a slide negative [16]. When the thick-smear was positive, species identification was done on thin blood films based on morphological criteria with the aid of identification tables as described by Cheesbrough [17].

#### 2.3.2. Widal Test

Typhoid fever was diagnosed using semiquantitative (rapid slide agglutination test) and quantitative methods (tube agglutination tests) Widal test as well as described by [6]. Slide agglutination test was done for typhoid fever screening using somatic (O) and flagellar (H) antigens kits of *S. typhi* (TYDAL, Lab Care Diagnostics, India). Antibody regents TO and TH were used for *S. typhi* detection while AO, BO, CO, AH, BH, and CH were used for *S. paratyphi* detection. Antibody titer of ≥1 : 80 against O and H antigen of *S. typhi* was considered as positive, and samples were subjected to standard tube agglutination test for the quantitative approach. The procedure was repeated twice for each of the positive samples.

### 2.4. Data Analysis

Descriptive analysis of all variables was performed for association of diseases (malaria, typhoid fever, coinfection) with sociodemographics characteristics and clinical features using chi‐square and Fisher's exact tests for significance at 95% probability level using XLStat™ v. 2016.02 software (Addinsoft, France).

### 2.5. Ethical Consideration

Research authorization was obtained from the University of Ngaoundéré, Biomedical Sciences Department and the ethical committee, respectively. Administrative authorization was obtained from Ngaoundéré Regional Health Office and Ngaoundéré Regional Hospital. Written informed consent was obtained from each of the volunteer study subjects or parents of children. Informed consent of each adult participant was obtained before blood sample collection.

## 3. Results

*Sociodemographics Data*. A total number of 208 febrile patients coming for the medical consultation at the Ngaoundéré Regional Hospital and suspected for malaria and/or typhoid fever were included in this study. About 57.7% of the study participants were females, and participants were aged between 1 and 70 years. The majority of the patients (38.5%) were within 2 to 10 years, most of them were single (71.2%), attended primary school (49.5%), lived in subrural areas (80.3%), and performed daily labors (22.6%) (Table 1).

**Figure 1:**
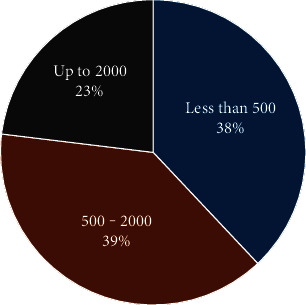
Repartition of parasitemia among febrile patients with malaria (*N* = 208).

*Prevalence of Malaria, Typhoid Fever and Coinfection*. The prevalence of malaria and typhoid fever among the participants is shown in Table 2. Globally, 79.3% (*n* = 165) of patients were infected with one of the diagnosed diseases. About 50.0% (104/208) were malaria positive, and *P. falciparum* were the only species identified. Parasitemia ranged from 200 to 4000 parasites/mm^3^, 39 (38.0%) subjects had less than 500 parasites/mm^3^, 41 (38.9%) ranging from 500 to 2000, and 24 subjects (23.1%) had up to 2000 parasites (Figure 1).

Typhoid fever was the most prevalent disease in the study area. A total of 133 patients (64.3%) had antibody titers of ≥1 : 80 for both O antigens. Twenty-four percent (*n* = 50) were positive at 1/320 titration level, 20.3% (*n* = 42) at 1/160, and 19.8% (*n* = 41) at 1/80 titration (Table 3).

About 63 (30.3%) patients were coinfected with malaria and typhoid fever. We also observed the presence of *Salmonella paratyphi* A, B, and C among the patients (*n* = 104) of the Ngaoundéré Regional Hospital with the prevalence of 62.0%, 62.5%, and 63.9%, respectively. The gender, educational level, and residence area were significantly associated with malaria prevalence, while the occupation and educational level were significantly associated with typhoid fever and the coinfection prevalence (*P* ≤ 0.05). For example, females were more prevalent (*n* = 77; 34.1%) to malaria than males (*n* = 33; 15.9%). In addition, residents of subrural area were more affected by malaria (90 patients, 43.3%). Among the educational group, subject with primary education background had the highest malaria prevalence (*n* = 61; 29.3%) while those in high education had the least (9, 4.3%). According to typhoid fever, the subjects of primary and secondary educational levels have the prevalence of 38.0% (*n* = 79) and 12.0% (*n* = 25), respectively. Ages 2–9 had the highest prevalence rate of malaria 22.1% (*n* = 46), typhoid fever 22.6% (*n* = 47), and the coinfection 12.5% (*n* = 26), but the association was not significant (*P* ≥ 0.05). There was no significant association of malaria and typhoid fever coinfection with age, sex, marital status, and residence (*P* > 0.05) (Table 4). Concerning the clinical features, fever and vomiting were significantly associated with malaria and typhoid fever (*P* = 0.00 and *P* = 0.01, respectively), while headaches, joint pain, vomiting, and diarrhea were significantly associated with the coinfection (Table 5). We noted on this study that treatment of either malaria or typhoid fever mainly consists of conventional medicine drugs used compared to the traditional medicine application (Table 6).

## 4. Discussion

Malaria and typhoid fever are still a major public health concern in tropics. The prevalence of malaria, typhoid fever, and coinfection is high in this study. They are tropical diseases where poverty, malnutrition, poor sanitary status, poor personal hygiene, poor health facilities, poor social service, and low levels of education are among the main risk factors [8].

### 4.1. Malaria

The present study works to provide information on concurrent malaria and typhoid infections in Ngaoundéré City. Speaking of malaria, globally, 79.3% (*n* = 165) of patients were infected with one of the diagnosed diseases. About 50.0% (104/208) of the patients were malaria positive, and *P. falciparum* was the only species identified. These results are concomitant with those reported by literature [15]. This result is comparable with those obtained by other authors with the rate of 55.5% and 54.2%, respectively, in Nigeria [6, 18]. However, it is less than the reports from Zambia (78.6%) [7] and higher than the reports from Fondenera, Cameroon (10.9%) [14], Calabar, Nigeria (26.7%) [9], and Northern Tanzania (24.2%) [11]. Females were more positive to malaria infection than males (*P* < 0.05), which is similar to the report of Sangaré et al. [10] in Burkina Faso, Odikamnoro et al. [18] in Nigeria, and Chilongola et al. [11] in Northern Tanzania. However, this result did not corroborate the report of Ubengama et al. [9] and Simon-Oke and Akinbote [8] who recorded a higher prevalence, respectively, among male students (30.9% and 43.0%) than female students (23.2% and 17.5%) in Calabar and Akure, Nigeria. In contrast, other authors reported that adults were most prevalent than young people [14]. The high prevalence recorded among this age group could be because of their different habits, level of exposure to mosquito bites, and immunity developed against the parasite. We noted a significant difference within the residence zone where rural area recorded the higher rate (90.0%). The discrepancy of the results between the studies might be due to seasonal variation and difference in geographical locations [14, 19, 20]. In addition, it could also been due to the presence of substantial gains in malaria program financing and coverage in specific geographical zones since the beginning of the millennium and that these have had a substantial impact on the incidence of malaria [14, 21], for example, the government malaria intervention program of distribution and use of Long Lasting Insecticidal Treated Nets (LLINs) in the rural communities of Cameroon and Ngaoundéré City particularly. The insecticide treated nets have had a significant impact in reducing the mortality rate of children under the age of 5 years by 20% as compared to those who use untreated nets [5]. The prevalence obtained in this study (50%) is well above the national prevalence. The increased malaria prevalence in the present work suggests the possibility of a breakdown of malaria control strategies in the area. In addition, the high value observed in this study compared to the value obtained (48 and 24%) in 2014 and 2018, respectively, in Cameroon could be explained by the high prevalence of multidrug-resistant parasites and insecticide-resistant malaria vectors, human migration of the population, high rate of deforestation, and lack of sanitation systems [4]. This might have resulted in the improvement in incidences of malaria and typhoid fever infection in Cameroon [15]. The age group 1–20 years is made up of children and students who have no knowledge of the implications of the bites received from the vectors of this disease; hence, they expose themselves to these vectors while sleeping [5]. In hyperendemic localities of *P. falciparum*, primary infections are seldom seen among adults but common in very young children and visitors [8, 20]. The predominance in these age groups might be due to low immune response against malaria infection, inappropriate use of bed nets, and inappropriate use of antimalarial drugs in the case of children [19]. On the other hand, the low prevalence among the age groups 41–50 years could be attributed to active immune response against these parasites and their careful habit in protecting themselves from the bites of the malaria vectors [20]. Authors reported that age group 2–5 years old was most associated with malaria prevalence [19, 20]. In Cameroon, authors reported that malaria accounts for 22% of deaths that occur in health care facilities in Cameroon [5].

### 4.2. Typhoid Fever

Typhoid fever prevalence rate in this study was about 64.3%PS. This data is lesser than the reports from Zambia (78.6%) [7] and higher than the reports in Fondenera, Cameroon (7.3%) [14], in Yola, Nigeria (39%) [6], in Calabar, Nigeria (43.3%) [9], and in Northern Tanzania (13.3%) [11]. Females were significantly (*P* = 0.00) more prevalent (34.1%; *N* = 77) than males (15.9%; *N* = 33). Similar observations have been reported in Burkina Faso [10] and in Nigeria [9]. However, the result did not corroborate the report of Simon-Oke and Akinbote [8] in Akure, Nigeria, recording a similar prevalence, respectively, among male students (40.5%) than female students (40.0%). The high prevalence of typhoid fever could be attributed to the source of drinking water (such as water from uncovered well, contaminated sachet water) and indiscriminate defecation in bushes around water sources which is a common practice among students which could have contributed to the high burden of typhoid fever recorded in this study [22–24]. It was interesting to know that young people (0–20 years) in this community were greatly infected with typhoid fever and this is in line with the report of Simon-Oke and Akinbote [8]. This can be explained by the fact that this age group is more active in this community and is involved in unhealthy activities like buying poorly prepared food from food vendors, drinking any available water, and travelling to endemic zones and back. The other sociodemographic parameter affecting the prevalence rate of typhoid fever is the level of education which is still low and prevalence was highest in primary educational level than any other levels. In addition, the lower occurrence was recorded among patients attended secondary and higher education, performed civil servants and merchants, and of 30–50 years old. This could be due to the fact that these groups have sufficient money and knowledge about the typhoid fever, have protection from infection, and have visited antenatal clinics more than people with little or no education [20]. In contrast, people with the level of education among illiterate and primary and secondary education groups particularly in rural area are still low since most of them still believe that Africans are resistant to infectious diseases and therefore consume food or water available to them irrespective of hygienic standards [20]. The medicinal plants for fevers may be because of poverty being low-income earners. Considering the treatment cost of both infections, most affected African countries, particularly the rural populations, are unable to cope with the burden of these diseases [25].

### 4.3. Coinfection

The prevalence of malaria and typhoid coinfection was 30.3%. Despite the low coinfection prevalence, there are important implications for the coinfected individuals. There was no significant association with the marital status, age groups, and residence area. However, we noted a significant association (*P* < 0.05) with education levels and profession with the high prevalence among the primary (64.2%) and secondary (20.3%) levels on the one hand and student (49.5%) on the other hand. These observations are in agreement with those reported by literature [14]. We found that females were more coinfected (35.1%; *N* = 76) than males (23.1%; *N* = 48), but not statistically significant (*P* > 0.05). This result did not corroborate the report of [8] in Akure, Nigeria, who recorded a higher prevalence, respectively, among male students (39.0%) than female students (12.5%). It is also important to note that in coinfection, the age group of 2–10 years was most prevalent. The mechanism to explain the association is not well established. However, literature reported that suffering from both complement deficiency and hemolysis during malaria infection can lead to iron deposition in the liver, further predisposing the patient to *Salmonella* sp. infection [26].

### 4.4. Clinical Features

Among the 208 participants, 38 (18%) responded positively for fever, 78 (37%) for chills, and 77 (37%) for vomiting. Also, 12 (5.8%) responded positively for headaches and 41 (19.7%) for fatigue. The similar clinical features have been mentioned in the literature [16]. Concomitant and intermittent fever was significantly associated with malaria; joint pain and vomiting were significantly associated with typhoid fever; headache, joint pain, vomiting, and diarrhea were significantly associated with malaria and typhoid fever coinfection (*P* < 0.05) (Table 4). These symptoms were confirmed as actual indication of malaria parasite presence [18].

## 5. Conclusion

Malaria and typhoid fever are two leading infections of poverty with serious health and socioeconomic impacts, and due to their geographical overlap, coinfections are very common. Coinfection rate was higher in females than in males, age group of 0–10 years recorded the highest prevalence, and some other risk factors such as profession and level of education were significantly affected by the diseases studied. Since they both have similar symptoms, treatment is based on adequate laboratory diagnosis. The data obtained in this study should be used in order to reduce mortality and morbidity caused by these infections in Ngaoundéré City.

## Figures and Tables

**Table 1:** Sociodemographic data of study subjects in Ngaoundéré Regional Hospital (*N* = 208).

Variable	Categories	*N*	Frequency (%)
Sex	Female	120	57.7
	Male	88	42.3

Age	[0-10]	80	38.5
	[10-20]	45	21.6
	[20-30]	39	18.8
	[30-40]	17	8.2
	[40-70]	27	13.0

Marital status	Single	148	71.2
	Married	57	27.4
	Widow(er)	3	1.4

Education	Illiterate	43	20.7
	Primary school	103	49.5
	Secondary school	42	20.2
	College/university	20	9.6

Residence	Subrural	167	80.3
	Urban	41	19.7

Occupation	Daily laborers	47	22.6
	Merchant	22	10.6
	Civil servant	9	4.3
	Housewife	37	17.8
	Student	93	44.7

**Table 2:** Distribution of malaria and typhoid fever among febrile patients (*N* = 207).

Infection	*N*	Frequency (%)
Malaria only	41	19.71
Typhoid only	61	29.33
Coinfection	63	30.29
Negative in both	43	20.67
Total	207	100

**Table 3:** Distribution of slide agglutination test results by titration (*N* = 208).

Widal titer	*N*	Frequency (%)
No agglutination	4	1.9
<1/80	70	33.8
1/80	41	19.8
1/160	42	20.3
1/360	50	24.2
Total	208	100

≥1 : 80 titer taken as positive titer.

**Table 4:** Prevalence (frequency in percentage (*N*)) of malaria, typhoid fever, and their coinfection in relation to sociodemographic characteristics.

Sociodemographic parameters	Categories	Malaria		*P* value	*χ* ^2^	Typhoid fever		*P* value	*χ* ^2^	Coinfection		*P* value	*χ* ^2^
		No	Yes			No	Yes			No	Yes		
Age	[0-10]	16.3 (34)	22.1 (46)	0.3	6.4	15.9 (33)	22.6 (47)	0.2	7.4	26.0 (54)	12.5 (26)	0.1	8.5
	[10-20]	13.5 (38)	8.2 (17)			8.2 (17)	13.5 (28)			16.3 (34)	5.3 (11)		
	[20-30]	8.7 (18)	10.1 (21)			7.7 (16)	11.1 (23)			11.1 (23)	7.7 (16)		
	[30-40]	5.3 (11)	2.9 (6)			4.3 (9)	3.8 (8)			7.7 (16)	0.5 (1)		
	[40-50]	5.3 (11)	5.3 (11)			2.4 (5)	8.2 (17)			6.7 (14)	3.8 (8)		
	>50	1.0 (2)	1.4 (3)			1.9 (4)	0.5 (1)			1.9 (4)	0.5 (1)		

Sex	Female	23.6 (49)	34.1 (77)	<0.01	9.5	21.2 (44)	36.5 (76)	0.2	1.6	34.6 (72)	23.1 (48)	12.7	0.0
	Male	26.4 (55)	15.9 (33)			19.2 (40)	23.1 (48)			35.1 (73)	7.2 (15)		

Marital status	Single	35.6 (74)	35.6 (74)	0.8	0.4	29.8 (62)	41.3 (86)	0.4	1.7	49.5 (103)	21.6 (45)	1.0	0.0
	Married	13.9 (29)	13.5 (28)			9.6 (20)	17.8 (37)			19.2 (40)	8.2 (17)		
	Widow(er)	0.5 (1)	1.0 (2)			1.0 (2)	0.5 (1)			1.0 (2)	0.5 (1)		

Education	Illiterate	10.6 (22)	10.1 (21)	<0.01	9.8	15.9 (33)	4.8 (10)	<0.01	36.8	7.2 (15)	2.4 (5)	<0.01	23.0
	Primary	20.2 (42)	29.3 (61)			11.5 (24)	38.0 (79)			19.2 (40)	1.4 (3)		
	Secondary	13.9 (29)	6.3 (13)			8.2 (17)	12.0 (25)			27.4 (57)	22.1 (46)		
	University	5.3 (11)	4.3 (9)			4.8 (10)	4.8 (10)			15.9 (33)	4.3 (9)		

Profession	Civil servant	1.9 (4)	2.4 (5)	0.3	9.5	1.4 (3)	2.9 (6)	<0.01	16.5	3.4 (7)	1.0 (2)	<0.01	19.2
	Daily laborers	12.5 (26)	10.1 (21)			14.4 (30)	8.2 (17)			20.2 (42)	2.4 (5)		
	Housewife	7.2 (15)	10.6 (22)			4.3 (9)	13.5 (28)			9.6 (20)	8.2 (17)		
	Merchant	7.2 (15)	3.4 (7)			4.8 (10)	5.8 (12)			9.1 (19)	1.4 (3)		
	Student	21.2 (44)	23.6 (49)			15.4 (32)	29.3 (61)			27.4 (57)	17.3 (36)		

Residence	Subrural	37.0 (77)	43.3 (90)	0.0	5.1	31.3 (65)	49.0 (102)	0.4	0.8	53.4 (111)	26.9 (56)	0.1	3.5
	Urban	13.0 (27)	6.7 (14)			9.1 (19)	10.6 (22)			16.3 (34)	3.4 (7)		

**Table 5:** Prevalence (frequency in percentage (*N*)) of malaria and typhoid fever infection in relation to clinical features in febrile patients.

Clinic	Categories	Malaria		*P* value	*χ* ^2^	Typhoid fever		*P* value	*χ* ^2^	Coinfection		*P* value	*χ* ^2^
		Yes	No			Yes	No			Yes	No		
Fever	Continuous	13.0 (27)	37.0 (77)	**<0.01**	**8.2**	7.2 (15)	33.2 (69)	**0.90**	**0.0**	14.9 (31)	3.4 (7)	**0.08**	**3.10**
	Intermittent	5.3 (11)	44.7 (93)			11.1 (23)	48.6 (101)			54.8 (114)	26.9 (56)		

Headaches	No	10.1 (21)	39.9 (83)	**0.09**	**2.9**	8.7 (18)	31.7 (66)	**0.07**	**3.3**	13.9 (29)	1.9 (4)	**<0.01**	**6.13**
	Yes	5.8 (12)	44.2 (92)			7.2 (15)	52.4 (109)			55.8 (116)	28.4 (116)		

Joint pain	No	13.5 (28)	36.5 (76)	**0.90**	**2.8**	12.0 (25)	28.4 (59)	**0.03**	**4.8**	20.2 (42)	1.9 (4)	**<0.01**	**13.04**
	Yes	8.7 (18)	41.3 (86)			10.1 (21)	49.5 (103)			49.5 (103)	28.4 (59)		

Fatigue	No	21.2 (44)	28.8 (60)	**0.67**	**0.2**	16.3 (34)	24.0 (50)	**0.96**	**0.0**	28.8 (60)	12.0 (25)	**0.82**	**0.05**
	Yes	19.7 (41)	30.3 (63)			24.5 (51)	35.1 (73)			40.9 (85)	18.3 (38)		

Chill	No	41.3 (86)	8.7 (18)	**0.2**	**1.8**	33.2 (69)	7.2 (15)	**0.34**	**0.9**	56.3 (117)	22.6 (47)	**0.32**	**0.98**
	Yes	37.5 (78)	12.5 (26)			45.7 (95)	13.9 (29)			13.5 (28)	7.7 (16)		

Vomiting	No	38.5 (80)	11.6 (24)	**0.15**	**3.8**	34.6 (72)	5.8 (12)	**<0.01**	**10.4**	55.8 (116)	19.7 (41)	**0.02**	**8.09**
	Yes	37.0 (77)	13.0 (27)			40.9 (85)	18.8 (39)			13.9 (29)	10.6 (22)		

Diarrhea	No	34.1 (71)	15.9 (33)	**0.60**	**1.0**	25.5 (53)	14.9 (31)	**0.31**	**2.3**	44.2 (92)	23.6 (49)	**0.03**	**7.14**
	Yes	33.7 (70)	16.4 (34)			42.3 (88)	17.3 (35)			25.5 (53)	6.7 (14)		

**Table 6:** Prevalence (frequency in percentage (*N*)) of malaria, typhoid fever, and coinfection in relation to medical history.

Treatment approach/response		Malaria		*P* value	*χ* ^2^	Typhoid fever		*P* value	*χ* ^2^	Coinfection		*P* value	*χ* ^2^
		No	Yes			No	Yes			No	Yes		
Treatment	**No**	13.5 (28)	10.6 (22)	**0.33**	**0.95**	8.7 (18)	15.4 (32)	**0.47**	**0.53**	15.9 (33)	8.2 (17)	**0.51**	**0.43**
	**Yes**	36.5 (76)	39.4 (82)			31.7 (66)	44.2 (92)			53.8 (112)	22.1 (46)		

Antimalarial	**No**	28.4 (59)	28.8 (59)	**0.59**	**1.05**	25.0 (52)	32.2 (67)	**0.27**	**2.60**	40.4 (84)	16.8 (35)	**0.77**	**0.51**
	**Yes**	21.6 (45)	21.2 (44)			15.4 (32)	27.4 (57)			29.3 (61)	13.5 (28)		

Antibiotics	**No**	47.1 (98)	45.2 (94)	**0.30**	**1.08**	35.1 (73)	57.2 (119)	**0.02**	**5.79**	63.0 (131)	29.3 (61)	**0.11**	**2.60**
	**Yes**	2.9 (6)	4.8 (6)			5.3 (11)	2.4 (5)			6.7 (14)	1.0 (2)		

Traditional medicine	**No**	49.5 (103)	50.0 (104)	**0.32**	**1.00**	40.4 (84)	59.1 (123)	**0.41**	**0.68**	69.2 (144)	30.3 (63)	**0.51**	**0.44**
	**Yes**	0.5 (1)	0.0 (0)			0.0 (0)	0.5 (1)			0.5 (1)	0.0 (0)		

## Data Availability

The data used to support the findings of this study are available from the corresponding author on request.
